# An Ultrasound‐Assisted *Pistacia terebinthus* L. Extract to Incorporate Into Fermented Sausage

**DOI:** 10.1002/fsn3.70853

**Published:** 2025-08-27

**Authors:** Hakan Benli, Özge Tetik

**Affiliations:** ^1^ Department of Food Engineering, Faculty of Engineering Cukurova University Adana Turkey

**Keywords:** biogenic amines, green extraction, natural antioxidant, phytochemicals, refrigerated storage, traditional sausage

## Abstract

In this study, the incorporation of varying levels of an ultrasound‐assisted *Pistacia terebinthus* L. extract into fermented sausages (sucuks) and their effects on the quality attributes and biogenic amine formation were investigated. Terebinth extracts were prepared using 90%, 80%, 70%, 60% ethanol and distilled water. Ultrasound‐assisted 70% ethanol extract was selected due to its higher total phenolic content and DPPH value. Five groups of experimental sucuks were produced by incorporating 1, 5, and 10 mL/kg of terebinth extract as a natural source of antioxidants and phytochemicals and adding 500 mg/kg of ascorbic acid and a control group. Terebinth extract incorporated groups had lower pH values in the end products when compared to control and ascorbic acid groups. The end products of all groups had moisture contents around 40%. Incorporating terebinth extract produced similar color values, texture profile analysis (TPA), and TBARS values when compared to control and ascorbic acid groups. Accumulation of biogenic amines (mg/kg dry weight) including tryptamine (9.21–30.50), 2‐ phenylethylamine (1.68–27.51), putrescine (1.20–5.84), cadaverine (2.47–5.79), histamine (6.50–8.08), tyramine (2.77–78.33), spermidine (4.00–6.84), and spermine (51.08–65.83) were in the expected ranges for fermented sausages. Even though terebinth extracts were not effective to decrease biogenic amine accumulation in the samples, there were no significant differences in biogenic amine contents of the end products. The current study demonstrated that terebinth extracts as a source of antioxidants and phytochemicals can be incorporated into fermented sausages without having a negative effect on the overall quality.

## Graphical abstract

A selected ultrasound‐assisted terebinth extract (70% ethanol extract) was incorporated into a fermented sausage (sucuk) at different levels (1, 5, and 10 mL/kg) without having a negative effect on the quality and biogenic amine accumulation when compared to ascorbic acid (500 mg/kg) and a control group. As a result, the terebinth extract could be incorporated into the commercial production of fermented sausages to improve their phytochemical profile and antioxidant properties.

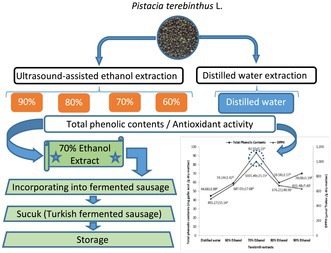

## 1 Introduction

Fermented sausages are popular meat products, valued for their unique flavor and texture (Abril et al. 2023). In general, fermented foods are recommended for enriching a diet to gain numerous beneficial health effects. However, one of the major concerns with fermented sausages is the formation of high concentrations of biogenic amines, which could endanger the well‐being of consumers (Swider et al. 2023). Sucuk is a traditional Turkish fermented sausage manufactured by combining ground beef, fat or sheep tail fat, curing agents (salt, nitrite, or nitrate), sugar, and a blend of spices including black pepper, red pepper, allspice, cumin, and garlic. The sucuk dough is stuffed into natural or synthetic casings. Then the fermentation is achieved either through inoculation with a starter culture or through natural fermentation. Following the fermentation, the product undergoes drying and ripening stages, which can be conducted under natural or controlled conditions. Semi‐dry or dry fermented sausages are the result of a traditionally manufactured Turkish sucuk (Benli 2017; Benli et al. 2024).

Spices and herbs can be incorporated into meat and meat products whole or as an extract (Yin and Cheng 2003). The extraction is intended to collect high‐quality compounds with a higher amount of antioxidants. Phytochemicals can be extracted using solvents from different parts of plants and utilized as natural antioxidants (Benli et al. 2024; Dai and Mumper 2010). Water and ethanol are considered green solvents since they are renewable, recyclable, biodegradable, and nontoxic. Water is a non‐selective and frequently used green solvent. However, water can extract all hydrophilic compounds, including polysaccharides, saponins, phenols, etc., due to its non‐selectivity. In contrast, ethanol is more selective in action and is usually utilized to extract polyphenols. Furthermore, mixing ethanol with water and assisting with ultrasound might increase the effectiveness of ethanol extraction. Ultrasound‐assisted extraction is utilized in a variety of food processes for extracting bioactive compounds from different plant parts (Benli et al. 2024; Kumar et al. 2023; Muniz‐Marquez et al. 2013). Ultrasound improves the ability of solvents to penetrate plant cells to increase the extraction yield since cavitation bubbles disrupt cell walls (Muniz‐Marquez et al. 2013). Ultrasound‐assisted extraction can be achieved by mixing the ground sample with an appropriate solvent and utilizing an ultrasonic bath in a laboratory setting while controlling temperature and extraction time (Altemimi et al. 2017).

*Pistacia terebinthus* L. (terebinth) is a plant native to the Mediterranean and Asia and belongs to the *Anacardiaceae* family. In Turkey, terebinth grows without cultivation in Eastern Anatolia, Central Anatolia, the inner parts of the Black Sea region, and the mountainous parts of the Mediterranean region. Terebinth is a broad and bushy tree with a rich resinous scent and glossy leaves. The terebinth fruits are small and spherical in shape and ripen between August and September. The plant, which has long been valued for its fragrant and therapeutic qualities, is high in tannin and resinous materials. The fruits are utilized as a folk remedy for rheumatism, gastritis, and coughs in addition to being used as a diuretic, anti‐rheumatic, antitussive, and stimulant (Kaya and Özer 2015; Ozcan 2004). Terebinth fruits are rich in phenolic compounds such as flavonoids and phenolic acids (Kamiloglu et al. 2022). 6′‐hydroxyhypolaetin 3′‐methyl ether was identified in the fruits in addition to several known flavonoids including apigenin, luteolin, luteolin 7‐*O*‐glucoside, quercetin, quercetagetin 3‐methyl ether 7‐*O*‐glucoside, and isoscutellarein 8‐*O*‐glucoside (Topçu et al. 2007). Terebinth fruits had a strong radical scavenging activity comparable to standards and recognizable metal‐chelation properties when compared to EDTA (Bozorgi et al. 2013). Similarly, Topçu et al. (2007) indicated that methanol extracts of terebinth fruits had a higher free radical scavenging activity and higher amounts of total phenolic and flavonoid contents when compared to the acetone extract. Akyuz et al. (2022) studied the antidiabetic properties of extracts obtained from different parts of the terebinth fruit. Terebinth fruits were reported to be rich in luteolin, and ethanol and acetone extracts obtained from terebinth fruits had the highest luteolin contents. Consumption of the terebinth fruits was suggested for their potential antidiabetic effect due to the strong inhibitory action of their extracts on the α‐glucosidase enzyme. Moreover, Bakırel et al. (2003) reported that feeding hypercholesterolemic rabbits with terebinth‐containing pellets lowered their blood lipid levels without any toxicological effect, limited the development of the atherosclerotic lesions in their thoracic artery, and caused fatty changes and hydropic degeneration in their liver.

Biogenic amines are low‐molecular‐weight organic compounds formed as byproducts of microbial metabolism in fermented foods. The decarboxylation of amino acids by microorganisms leads to the production of biogenic amines with various chemical structures, categorized as aliphatic, aromatic, or heterocyclic. Amino acid decarboxylases, enzymes responsible for the decarboxylation of amino acids, are present in many microorganisms, including the members of *Micrococcaceae, Pseudomonadaceae*, *Enterobacteriaceae*, and lactic acid bacteria (Ruiz‐Capillas and Herrero 2019; Wang et al. 2015; Xu et al. 2023). Biogenic amines are generated from corresponding amino acids by removing the α‐carboxyl group in the cytoplasm of decarboxylase‐producing microorganisms. Agmatine, histamine, cadaverine, phenylethylamine, tyramine, and tryptamine are directly produced from free amino acid precursors including arginine, histidine, lysine, phenylalanine, tyrosine, and tryptophan, respectively. While putrescine can be directly formed from ornithine, it can also be produced from arginine, glutamine, or agmatine. In addition, spermine and spermidine synthase enzymes transform putrescine into polyamines of spermine and spermidine, respectively (Swider et al. 2023). Histamine, cadaverine, putrescine, tryptamine, tyramine, β‐phenylethylamine, spermine, and spermidine were reported among the most significant biogenic amines found in foods (Sun et al. 2020). Microorganisms seem to synthesize biogenic amines due to their physiological role in the defense mechanisms to tolerate acidic environments (Rhee et al. 2002). Decarboxylation aids bacteria in restoring the internal pH to survive in acidic environments by consuming protons and excreting CO_2_ and amines (Lee et al. 2007; van de Guchte et al. 2002). In addition, bacteria with amino acid decarboxylase activity could tolerate or decrease the effects of stress‐induced cell responses, including oxygen and NaCl, via producing biogenic amines (Spano et al. 2010).

The quantity of biogenic amines in foods is important due to their consideration as a quality indicator and their possible toxicity to human health (Ruiz‐Capillas and Herrero 2019). Higher amounts of biogenic amines from diet‐related sources may be harmful to the nervous and cardiovascular systems, as well as potentially cause neurological headaches and arrhythmia (Li et al. 2023). The formation of biogenic amines could be reduced using two different approaches in fermented foods, including inhibiting the growth of microorganisms and inhibiting the amino acid decarboxylase activity of microorganisms, although the first approach might result in losing the authentic flavor of the fermented products (Lee et al. 2018). Wendakoon and Sakaguchi (1995) indicated that decarboxylase activity of microorganisms can be inhibited with ethanol extracts of certain spices, including cloves, cinnamon, sage, nutmeg, and allspice. Wang, Liu, et al. (2022) reported that the incorporation of plant‐derived natural ingredients and extracts containing phenolic compounds, alkaloids, terpenes, and other antibacterial compounds was examined in recent studies to decrease the formation of biogenic amines in meat products. Furthermore, phenolic compounds were stated to decrease putrescine formation by preventing the cells from oxidative damage via their antioxidant effects (Alberto et al. 2007). In addition, fermented sausages are known to be vulnerable to lipid oxidation due to their high fat content and certain manufacturing techniques, including grinding and mixing. Natural substances, such as plant extracts, could also be used to reduce lipid oxidation and enhance the preservation of foods due to their antioxidant properties (Manzoor et al. 2023). Although incorporating plant extracts was reported to have a potential negative impact on the quality of the food products because of their distinctive color and aroma (Plaskova and Mlcek 2023), to the best of our knowledge, the incorporation of terebinth extracts into fermented sausages was not extensively studied in the literature. Thus, the purpose of the current study was to incorporate varying levels of an ultrasound‐assisted *Pistacia terebinthus* L. extract into Turkish fermented sausages and evaluate their quality properties and biogenic amine formations during the manufacturing and storage.

## 2 Materials and Methods

Terebinth ( *Pistacia terebinthus* L.) fruits, harvested from naturally grown trees in the district of Mansurlu in Adana, Turkey (37°52′05.9″ N 35°37′25.6″ E) were purchased from a local supplier. Beef (80% lean meat and 20% fat) was obtained from a meat manufacturing plant located in the district of Havutlu in Adana, Turkey. Fermented sausages (sucuks) were produced in a Pilot Sucuk Plant at Cukurova University. All chemicals utilized in this study were of analytical grade.

### 2.1 Production of Terebinth Extracts

Five different terebinth extracts were produced using 90%, 80%, 70%, and 60% ethanol solutions and distilled water to determine an extract with a higher antioxidant capacity to incorporate into fermented sausages. Terebinth fruits were pulverized in a blender. Equal amounts of powdered terebinth (25 g) were weighed and added to 250 mL of 90%, 80%, 70%, and 60% ethanol solutions, respectively. The mixtures were then placed in an ultrasonic bath (Bandelin Sonorex RK 1028 H, Bandelin Electronic, Berlin, Germany) for an hour at room temperature (Benli et al. 2024; Kurcubic et al. 2014). The same amount of powdered terebinth (25 g) was also weighed and added to 250 mL of distilled water and placed in a water bath (Memmert, WNB22, Schwabach, Germany) with continuous shaking for 24 h (Benli et al. 2024; Bozkurt 2006). All extracts were filtered using coarse filter papers. Then, each extract was concentrated to approximately 50 mL using a rotary evaporator (Hei‐VAP Advantage Rotary Evaporator, Heidolph Instruments, Schwabach, Germany) under vacuum at 40°C. Each terebinth extract was prepared three times and combined. The extracts were then analyzed for antioxidant activity and total phenolic content to determine their antioxidant capacity.

### 2.2 Total Phenolic Contents of Terebinth Extracts

The extracts obtained from terebinth were mixed with 80% methanol (1:10 *v/v*), separately, and placed in a Hettich Zentrifugen Mikro 220R (Andreas Hettich GmbH & Co., Tuttligen, Germany) centrifuge at 4°C for 20 min at 4000 rpm. After transferring the supernatants (100 μL) into glass tubes, 100 μL Folin–Ciocalteu solution and 3000 μL distilled water were added to each glass tube and mixed. Distilled water (100 μL) was used as a control. Then, all glass tubes were incubated for 10 min. Next, 100 μL of 20% Na_2_CO_3_ (*w/v*) solution was added to the glass tubes. The mixtures were further incubated in a dark place for 2 h before taking absorbance values at 765 nm in a Lambda 25 UV/VIS Spectrometer (Perkin Elmer, Connecticut, USA). Gallic acid solutions with different concentrations were used to prepare a calibration curve for calculating total phenolic contents of the extracts. The total phenolic contents of the extracts were calculated as gallic acid equivalent/g dry weight (Abdullakasim et al. 2007; Agcam et al. 2021).

### 2.3 Antioxidant Activity of Terebinth Extracts

A previously described procedure for DPPH (1,1‐diphenyl‐2‐picrylhydrazyl) free radical‐scavenging method with slight modifications was used to assess antioxidant activities of terebinth extracts (Agcam et al. 2021; Klimczak et al. 2007). The extracts obtained from terebinth were mixed with 80% methanol (1:20 v/v), separately, and placed in a Hettich Zentrifugen Mikro 220R (Andreas Hettich GmbH & Co., Tuttligen, Germany) centrifuge at 4°C for 20 min at 4000 rpm. After transferring the supernatants (100 μL) into glass tubes, 2460 μL DPPH* radical (0.050 g/L in a solution of 80% methanol) were added to each glass tube and mixed using a vortex. Distilled water (100 μL) was used as a control. All tubes were kept in a dark place to equilibrate for 1 h before taking absorbance values at 515 nm in a Lambda 25 UV/VIS Spectrometer (Perkin Elmer, Connecticut, USA). The results were reported as μmol Trolox equivalent/g dry weight.

### 2.4 Manufacturing of Fermented Sausages

Sucuks were manufactured in Cukurova University Pilot Sucuk Plant. Five experimental groups of sucuks were produced. Ultrasound‐assisted 70% ethanol extract of terebinth was selected in the first phase of the study to incorporate into three experimental sausage groups with levels of 1 mL/kg (T‐1), 5 mL/kg (T‐5), and 10 mL/kg (T‐10). Ascorbic acid (500 mg/kg) was added to one experimental group while the control group was manufactured without any antioxidant addition. To prepare the sausage dough, 80% lean beef and 20% fat were ground using a 6 mm grinder plate. First, a starter culture mix ( *Staphylococcus carnosus* ve *Latilactobacillus sakei*) was added to the sausage dough according to the manufacturer's instruction. Next, 7 g of ground red pepper, 9 g of cumin, 5 g of black pepper, 2.5 g of allspice, 10 g of fresh garlic, 2 g of sucrose, 0.15 g of sodium nitrite, and 17.5 g of salt were added into the dough for each kilogram of meat base. Five equal batches of sausage dough were prepared, following adequate mixing. Then, four batches were randomly assigned to incorporate three levels of the terebinth extract and the ascorbic acid, while the last group was allocated as the control. After incorporating, all batches were ground using a 3 mm grinder plate and stuffed into natural casings (44 mm in diameter) to prepare a total of 75 sucuks (approximately 150 g each). Following the hanging, sucuks were subjected to fermentation for 3 days in a fermentation cabinet. The fermentation process was carried out at 85%–90% relative humidity and 22°C ± 1°C on the first day, 85%–90% relative humidity and 20°C ± 1°C on the second day, and 80%–85% relative humidity and 18°C ± 1°C on the third day. Then, the temperature was gradually reduced to below 16°C while the relative humidity was gradually reduced to 70% for drying for 4 days. Finally, sucuks were vacuum‐packed and kept for 60 days at 4°C. Samples were taken prior to fermentation, following fermentation, from the final product, 30 days after storage, and 60 days after storage for the analyses. Two replications of the entire experiment were conducted.

### 2.5 Moisture Content, pH, and Titratable Acidity of Fermented Sausages

Samples were dried in a Memmert Universal Oven (Schwabach, Germany) at 100°C for 16 h according to the oven drying method to determine moisture contents of sucuk samples (Nielsen 2003). A calibrated pH meter (S220, Mettler‐Toledo LLC, Columbus, OH, USA) was used to measure the pH values in slurries obtained by mixing 5 g of samples with 45 milliliters of distilled water (Ockerman 1985). Next, 0.1 N NaOH was used to titrate each sample to determine the titratable acidity of the samples. The results were reported as percent lactic acid (Gökalp et al. 2001).

### 2.6 Color Values of Fermented Sausages

Color values were obtained from the inner surfaces of the samples, including L* (lightness), a* (redness), and b* (yellowness). A standard white tile (*Y* = 93.7, *x* = 0.3157, *y* = 0.3323) was used to calibrate the colorimeter (Chroma Meter CR‐400, Konica Minolta Sensing Inc., Japan). The colorimeter had an 8 mm aperture size and was configured for a 2° observer with D‐65 illumination. The inner surface of each sucuk sample was measured in three different locations (Benli et al. 2024; Calnan et al. 2016).

### 2.7 Texture Profile Analysis (TPA) of Fermented Sausages

Texture Profile Analysis (TPA) parameters (hardness, adhesiveness, cohesiveness, chewiness, and resilience) were measured using a texture analyzer (Model TA–XT Plus, Stabile Microsystems, England). Three sucuk samples were prepared with a thickness of 1 cm and a diameter of 3 cm. A double compression cycle was used to compress the samples vertically to 75% of their initial height. The testing conditions were pre‐test speed of 3.0 mm/s, test speed of 1.0 mm/s, and post‐test speed of 3.0 mm/s. A 50 mm cylindrical probe and a 50 kg load cell were attached to the texture analyzer (Benli et al. 2024; Kargozari et al. 2014).

### 2.8 Biogenic Amine Contents of Fermented Sausages

Biogenic amine contents of the sucuk samples were determined according to a method described by Eerola et al. (1993) and Gençcelep et al. (2008) with slight adjustments (Benli et al. 2024). Following the extraction of biogenic amines from the samples, dansyl derivatives of the biogenic amines were determined using HPLC/PDA. Twenty milliliters of 0.4 M perchloric acid were transferred on 2 g of sample. An Ultra‐Turrax homogenizer (T‐18 digital Ultra‐Turrax, IKA Works GmbH & Co. KG, Staufen, Germany) was used to homogenize the sample for 60 s at 8000 rpm. After centrifuging for 10 min at 3000 rpm, the extract was filtered using filter paper, and a 0.4 M perchloric acid solution was added to complete the extract to 50 mL. Solutions of 2 N NaOH (200 μL) and saturated NaHCO_3_ (300 μL) were transferred on 1 mL of the extract for derivatization of the samples. Following the addition of 2 mL of dansyl chloride (10 mg/mL in acetone, w/v), the samples were placed in an oven (Memmert, Universal Oven Tech., Germany) at 40°C for 45 min for incubation. Then, 25% of ammonia (100 μL) was transferred and acetonitrile was used to complete the total volume of the samples to 5 mL. A centrifuge (Hettich Zentrifugen Mikro 220R, Andreas Hettich GmbH & Co., Tuttligen, Germany) was utilized for centrifuging the samples at 3000 rpm for 5 min. Next, the samples were filtered using a 0.45 μm PTFE filter. To create standard curves, perchloric acid solution (0.4 M) was added to complete biogenic amine standards to 1 mL, and the same procedure previously described for the samples was also applied for the derivatization of the standards. The mobile phases contained 0.1 M ammonium acetate (solvent A) and acetonitrile (solvent B). Gradient elution of the mobile phase included at the beginning (50% A + 50% B), at 25 min (10% A + 90% B), and at 35 min (50% A + 50% B). The flow rate was 1 mL/min, and the temperature of the column (Nukleodur 100–5‐C18; 12.5 × 4 mm) was 40°C. The column was injected with the volume of 20 μL of the samples. The measurements were taken at a wavelength of 254 nm. The components of the HPLC system included a photodiode array detector (SPD‐M20A), a column oven (CTO‐10AS), a computer running the Shimadzu LCsolution package application, a vacuum degasser (DGU‐20A5), a quaternary pump (LC‐20AT), an auto sampler (SIL‐20A), and an HPLC (Shimadzu, Japon). The current study examined the levels of biogenic amines, including tryptamine, 2‐phenylethylamine, putrescine, cadaverine, histamine, tyramine, spermidine, and spermine. Comparing the spectral data and retention times of the certified commercial standards allowed for the identification of the peaks. Five distinct concentrations of the standards were used for the calibration curves (*R* ^*2*^ = 0.997–0.999) from which the concentrations of the peaks were determined.

### 2.9 Thiobarbituric Acid Reactive Substances (TBARS) of Fermented Sausages

TBARS values of the sucuk samples were analyzed according to a method described by Mielnik et al. (2006) with slight adjustments (Benli et al. 2024). Thirty milliliters of an aqueous solution of trichloroacetic acid (7.6%) and 5 g of sample were homogenized with an Ultra‐Turrax homogenizer (T‐18 digital Ultra‐Turrax, IKA Works GmbH & Co. KG, Staufen, Germany) for 60 s at 9500 rpm. Following centrifugation for 20 min at 3000 rpm, the homogenate was filtered with a vacuum filter (Whatman No. 1 filter). Then, 5.0 mL of the extract was pipetted into a stoppered glass tube, and 0.02 M aqueous thiobarbituric acid (5 mL) solution was added. The glass tubes were placed in a water bath for 35 min at 100°C and cooled with tap water. Measurements of absorbance were performed at 532 nm using a spectrometer (Lambda 25 UV/VIS Spectrometer, Perkin Elmer, Connecticut, USA) with a blank sample (5 mL of distilled water and 5 mL of thiobarbituric acid solution). The TBARS values were calculated as mg malondialdehyde/kg of sausage using a standard curve created with 1,1,3,3 tetraethoxypropane standard.

### 2.10 Statistical Analyses

Data were statistically analyzed using IBM SPSS Statistics 20 software (Armonk, NY, USA). In a completely randomized design, the analysis of variance (One‐way ANOVA) method was used to identify significant differences (*p* < 0.05) for physicochemical values. The Duncan multiple comparison test was performed when a significant difference was observed (Ott and Longnecker 2001).

## 3 Results and Discussion

### 3.1 Total Phenolic Content and DPPH Values of Terebinth Extracts

**FIGURE 1:**
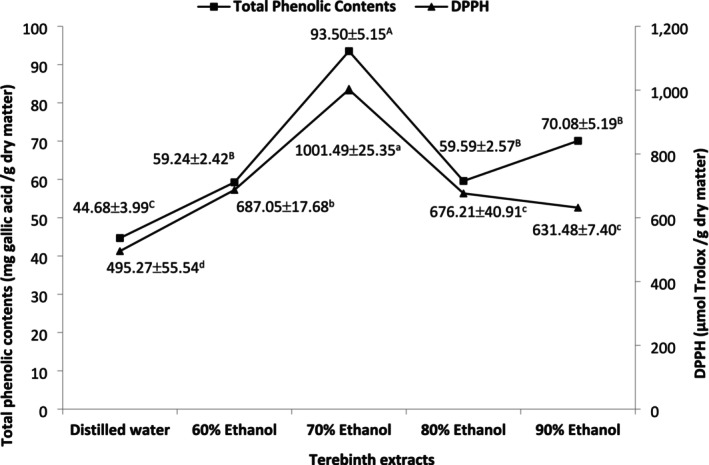
The total phenolic contents and DPPH values related to five different terebinth extracts. There are significant differences (*p* < 0.05) between values indicated with capital letters and between values indicated with lowercase letters for total phenolic contents and antioxidant activities of the extracts, respectively.

Figure 1 shows total phenolic contents and DPPH values of five terebinth extracts obtained using 90%, 80%, 70%, and 60% ethanol solutions and distilled water. The total phenolic content (93.50 mg gallic acid/g dry weight) and DPPH value (1001.49 μmol Trolox/g dry weight) were significantly higher with the ultrasound‐assisted extraction using 70% ethanol solution (*p* < 0.05). The distilled water extraction produced the lowest total phenolic content (44.68 mg gallic acid/g dry weight) and DPPH values (495.30 μmol Trolox/g dry weight). Furthermore, the total phenolic content and DPPH values of five terebinth extracts showed a positive correlation (*r* = 0.936, *p* < 0.05) in the current study. Phenolic compounds are synthesized by plants as secondary metabolites during their normal metabolisms and in reaction to stress conditions. These bioactive compounds have strong antioxidant properties and might help human beings to prevent various health issues (Haminiuk et al. 2014; Naczk and Shahidi 2006). The extraction process is expected to produce high‐quality phenolic compounds with higher levels of antioxidant properties. Various solvents including acetone, methanol, propanol, dimethylformamide, ethyl acetate, ethanol, and water were mostly used to extract phenolic compounds from different plant parts. However, water and ethanol are considered among the green solvents since they are nontoxic, recyclable, biodegradable, and renewable (Alothman et al. 2009; Altemimi et al. 2017; Kumar et al. 2023). Moreover, ethanol has been shown to have a selective activity to extract polyphenols with penetrating plant cell walls efficiently. By combining ethanol with water, the extraction efficiency of ethanol can be further increased (Kumar et al. 2023; Muniz‐Marquez et al. 2013). In addition, ultrasound‐assisted extraction was indicated to significantly enhance the extraction yield and efficiency of polyphenols due to mainly cavitation bubbles as well as erosion, fragmentation, sonoporation, local shear stress, sonocapillary effect, destruction–detexturation of plant materials (Chemat et al. 2017). Similarly, the highest total phenolic content and DPPH value were obtained using ultrasound‐assisted 70% ethanol extraction in the current study. Thus, the fermented sausages (sucuks) were manufactured with the incorporation of different levels of ultrasound‐assisted 70% ethanol extract in the study.

### 3.2 Moisture Values of Fermented Sausages

**FIGURE 2:**
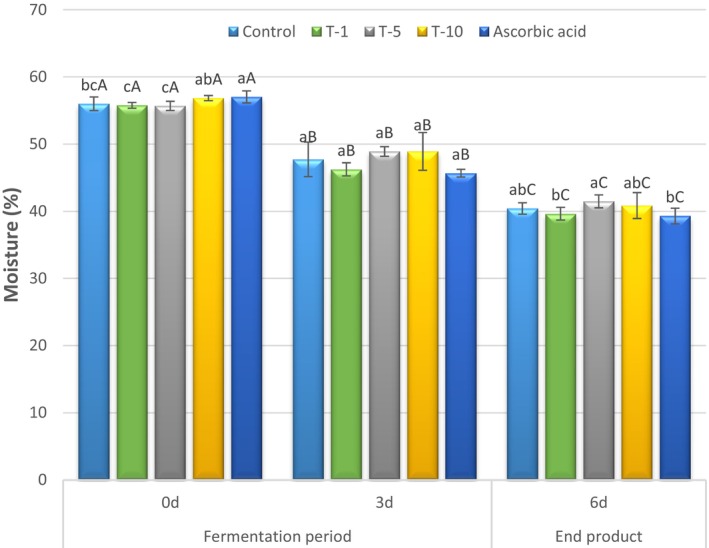
Mean moisture (%) contents of sucuks (fermented sausages) manufactured with different levels of terebinth extracts during production. Bars indicate standard deviation. There are significant differences between values represented with different lowercase letters on the same day (*p* < 0.05). There are significant differences between values represented with different capital letters for each treatment on different days (*p* < 0.05). T‐1: Included 1 mL/kg terebinth extract; T‐5: Included 5 mL/kg terebinth extract; T‐10: Included 10 mL/kg terebinth extract; and Ascorbic Acid: Included 500 mg/kg ascorbic acid.

Figure 2 represents mean moisture values of fermented sausages (sucuks). The effect of the treatments was significant on the moisture values of the initial and end products (*p* < 0.05). The mean moisture values ranged from 55.68% to 57.03% in the initial products (Day 0) while the end products had the mean moisture values ranging from 39.29% to 41.48% following the fermentation and drying. A traditional fermented sucuk was expected to have a moisture content of less than 40%, even though a higher variation ranging from 20.96% to 50.49% for moisture content was reported for sucuk samples collected from various regions in Turkey, indicating a lack of standardized manufacturing method in the industry (Benli 2017; Benli and Barutcu 2021; Benli et al. 2024; Siriken et al. 2009). In the current study, the end products had moisture contents around 40% as expected. Although statistical differences were observed in the moisture contents of the initial and end products, these variations could be considered within a limited range for a traditionally manufactured fermented sausage. Furthermore, the moisture contents declined significantly in all treatments throughout the manufacturing of sucuks (*p* < 0.05) due to the water evaporation that occurred during the fermentation and drying. In addition, organic acid accumulation, as a result of the fermentation, can lead to the denaturation of muscle proteins and the contraction of muscle fibers, ultimately contributing to the loss of water from the myofibrillar network (Zhang et al. 2024).

### 3.3 pH Values and Titratable Acidity Values of Fermented Sausages

**FIGURE 3:**
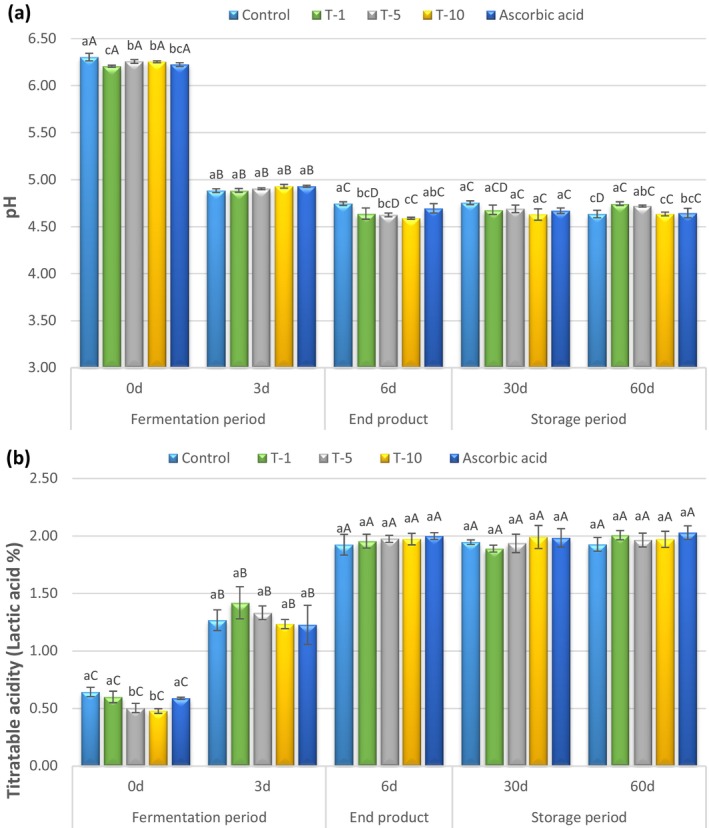
Mean pH (a) and titratable acidity (b) values of sucuks (fermented sausages) manufactured with different levels of terebinth extracts during production and storage. Bars indicate standard deviation. There are significant differences between values represented with different lowercase letters on the same day (*p* < 0.05). There are significant differences between values represented with different capital letters for each treatment on different days (*p* < 0.05). T‐1: Included 1 mL/kg terebinth extract; T‐5: Included 5 mL/kg terebinth extract; T‐10: Included 10 mL/kg terebinth extract; and Ascorbic Acid: Included 500 mg/kg ascorbic acid.

Figure 3 represents mean pH and titratable acidity values of fermented sausages (sucuks). As anticipated, all sucuk samples exhibited a significant decrease in pH values accompanied by an increase in titratable acidity (*p* < 0.05) during the fermentation period from Day 0 to Day 3. Manufacturing of a fermented sausage requires lactic acid bacteria to convert carbohydrates during the fermentation period to accumulate lactic acid. The accumulation of lactic acid leads to a decrease in pH and an increase in titratable acidity values in the fermented sausages. The acidification causes coagulation of the solubilized muscle proteins to form a gel around meat and fat particles and contributes to firmness. Thus, the hardness of a fermented sausage progressively increases while the pH declines from 5.4 to 4.9 (Bover‐Cid et al. 2001; Demeyer and Stahnke 2002). The average initial pH and titratable acidity values of sucuks ranged from 6.21 to 6.30 and from 0.48% to 0.65%, respectively. Following the fermentation, pH values decreased to a range of 4.88–4.93 while titratable acidity values increased to a range of 1.23%–1.42%. However, there were no significant differences in pH or titratable acidity values across the treatments. Nevertheless, the treatments had significant effects on pH values of Day 0, end products and Day 60 samples. The control had the highest pH values of 6.30 and 4.75 for the initial and end product, respectively. T‐1, T‐5, and T‐10 had the lowest pH values (4.64, 4.63 and 4.59, respectively) for the end product. Moreover, slight fluctuations observed in pH values were in a limited range during the storage. pH values of the treatments varied from 4.63 to 4.76 and from 4.64 to 4.75 on the storage Day 30 and Day 60, respectively. In addition, all treatments had similar titratable acidity values ranging from 1.89% to 2.03% in the end product and during the storage (Day 30 and Day 60). Conversely, Erkmen and Bozkurt (2004) stated that the pH values of 50 sucuk samples ranged from 4.53 to 6.74 in a study that examined sucuk samples from local markets and butchers in the Gaziantep region in Turkey. Gençcelep et al. (2008) analyzed 30 sucuk samples collected from butchers and local markets across multiple cities and reported that pH values ranged between 4.53 and 6.29. In another study regarding some chemical characteristics of sucuks collected from retail stores in Adana province in Turkey, Benli (2017) observed that the pH values of 36 sucuk samples varied highly, ranging from 4.69 to 6.56. Similarly, Benli and Barutcu (2021) indicated that pH values were highly variable (4.64–6.50) among 60 sucuk samples collected from Adana province. Although the majority of the research related to sucuks reported highly variable pH values due to a lack of standardized manufacturing methods, in the current study, incorporating an ultrasound‐assisted terebinth extract produced pH values with slight fluctuations.

### 3.4 Color Values of Fermented Sausages

Table 1 represents mean L*, a*, b*, chroma (C*), and hue (h*) values of fermented sausages (sucuks). In the current study, the control had a lower L* value (52.14) in the end product (*p* < 0.05) when compared to terebinth extracts and ascorbic acid added groups (53.82–55.10). Significant increases were observed in L* values of T‐5 and T‐10 on Day 60 of the storage (*p* < 0.05). However, all of the treatments had similar L* values on Day 30 and Day 60 of the storage ranging from 53.49 to 56.99. None of the treatments had a significant effect on a* and b* values in the end product (14.22–14.98 and 13.59–14.49, respectively), on Day 30 (11.93–13.07 and 12.43–13.11, respectively) and Day 60 (10.22–11.67 and 11.88–12.56, respectively) of the storage. But, overall a* and b* values indicated a tendency to decrease in redness and yellowness throughout the storage period. Furthermore, all of the treatments had similar C* values in the end product and on each of the storage days (Day 30 and Day 60) even though C* values of all treatments declined during the storage. The control had a lower h* value (redder appearance) and T‐10 had a higher h* value (yellowish or brownish tones) in comparison with the other treatments in the end product (*p* < 0.05). In addition, h* values indicated a tendency to increase throughout the storage period. However, all of the treatments had similar h* values on Day 30 and Day 60 of the storage. Even though incorporating the terebinth extracts seemed to have a slight effect on L* and h* values in the end products, all of the color values were similar during the storage period. Meat color is often associated with freshness, safety, and overall quality. Consumers evaluate a variety of food options, relying on the quality cues. The color is usually prioritized by consumers over the other quality cues to make a purchase decision for meat products. The quality cues are categorized into extrinsic and intrinsic cues. Extrinsic cues include external information unrelated to the physical attributes of food directly. However, intrinsic cues are related to inherent physical attributes of a product. Consumers utilize both intrinsic and extrinsic cues to form expectations about the overall eating quality of a product. In the case of beef, the most significant intrinsic quality indicators include color, marbling, and fat content, while origin and place of purchase are identified as the most influential extrinsic factors (Becker 2002; Benli et al. 2024). Since the color is considered one of the most significant intrinsic cues for the purchase decision, the consumer acceptability of sucuks might not be affected by incorporating terebinth extracts due to the limited color variation in samples when compared to the control and ascorbic acid groups.

**TABLE 1:** Mean color values of sucuks (fermented sausages) manufactured with different levels of terebinth extracts during storage.

Color	Storage period	Control	T‐1	T‐5	T‐10	Ascorbic acid
Lightness (L*)	End product	52.14 ± 0.77^bA^	53.82 ± 0.00^aA^	55.10 ± 0.42^aB^	54.79 ± 0.7^aB^	54.58 ± 0.69^aA^
	30 days	54.43 ± 0.25^aA^	54.25 ± 1.36^aA^	54.42 ± 0.20^aB^	53.49 ± 0.3^aB^	54.81 ± 1.73^aA^
	60 days	54.69 ± 0.88^aA^	54.63 ± 0.05^aA^	56.26 ± 0.76^aA^	56.99 ± 1.4^aA^	54.62 ± 0.67^aA^
Redness (a*)	End product	14.60 ± 0.60^aA^	14.67 ± 0.17^aA^	14.49 ± 0.30^aA^	14.22 ± 0.52^aA^	14.98 ± 0.09^aA^
	30 days	12.75 ± 0.35^aB^	12.74 ± 0.03^aB^	11.93 ± 0.51^aB^	12.66 ± 0.35^aB^	13.07 ± 0.41^aB^
	60 days	10.81 ± 0.13^aC^	11.67 ± 0.44^aC^	10.85 ± 0.64^aB^	10.22 ± 0.84^aC^	11.18 ± 0.49^aC^
Yellowness (b*)	End product	13.59 ± 0.66^aA^	14.08 ± 0.16^aA^	13.59 ± 0.12^aA^	13.95 ± 0.55^aA^	14.49 ± 0.26^aA^
	30 days	12.68 ± 0.35^aA^	12.43 ± 0.25^aB^	12.61 ± 0.21^aB^	12.52 ± 0.23^aA^	13.11 ± 0.35^aB^
	60 days	12.05 ± 0.69^aA^	12.44 ± 0.35^aB^	12.56 ± 0.33^aB^	12.31 ± 0.78^aA^	11.88 ± 0.19^aC^
Chroma (C*)	End product	19.95 ± 0.89^aA^	20.33 ± 0.24^aA^	19.86 ± 0.30^aA^	19.92 ± 0.76^aA^	20.18 ± 0.11^aA^
	30 days	17.98 ± 0.50 ^aAB^	17.80 ± 0.20^aB^	17.36 ± 0.38^aB^	17.81 ± 0.40^aB^	18.52 ± 0.04^aB^
	60 days	16.19 ± 0.42^aB^	17.06 ± 0.56^aB^	16.60 ± 0.17^aB^	16.03 ± 0.07^aC^	16.32 ± 0.47^aC^
Hue (h*)	End product	42.94 ± 0.23^cB^	43.83 ± 0.01^abcB^	43.18 ± 0.33^bcC^	44.45 ± 0.08^aA^	44.05 ± 0.69^abA^
	30 days	44.83 ± 0.01^aAB^	44.29 ± 0.52^aB^	46.59 ± 1.30^aAB^	44.69 ± 0.39^aA^	45.11 ± 1.67^aA^
	60 days	48.06 ± 1.98 ^aA^	46.82 ± 0.25^aA^	49.18 ± 2.42^aA^	50.28 ± 4.10^aA^	46.76 ± 0.80^aA^

*Note:* There are significant differences between the mean values (±standard deviation) represented with different lowercase letters on the same row (*p* < 0.05). There are significant differences between the mean values (±standard deviation) represented with different capital letters on the same column for each color parameters (*p* < 0.05). T‐1: included 1 mL/kg terebinth extract; T‐5: included 5 mL/kg terebinth extract; T‐10: included 10 mL/kg terebinth extract; and Ascorbic Acid: included 500 mg/kg ascorbic acid.

### 3.5 TPA Values of Fermented Sausages

Table 2 represents mean hardness, adhesiveness, cohesiveness, chewiness, and resilience values of fermented sausages (sucuks). Texture is an important indicator of sensory quality in meat products, impacting consumer acceptance and preference. Since texture is often associated with overall product quality, it plays an important role in purchasing decisions and eating satisfaction (Cardona et al. 2023). In the current study, incorporating terebinth extracts produced similar hardness, adhesiveness, cohesiveness, chewiness, and resilience values to the control and ascorbic acid group in the end product and during storage, except adhesiveness and cohesiveness values on Day 60. Hardness was defined as an amount of force needed to compress a sample similar to biting it down evenly between molar teeth. Storage caused significant increases in hardness values of T‐1, T‐10, and ascorbic acid groups (*p* < 0.05). Furthermore, hardness values of all treatments indicated a tendency to increase numerically with storage. Thus, sucuks might become harder to bite during storage, regardless of the addition of terebinth extracts. Adhesiveness was defined as the work required to overcome the attractive forces between the sample surface and a contact surface, similar to pulling food away from teeth or tongue, indicating stickiness. All treatments had similar adhesiveness values in the end product and on Day 30 of storage. However, T‐5, T‐10, and ascorbic acid treatments might become stickier when compared to the control and T‐1 on Day 60 of storage (*p* < 0.05). Even though numerical increases were observed with storage, the effect of storage was only significant on adhesiveness values of T‐1 (*p* < 0.05). Cohesiveness was the degree of a sample's resistance to breaking under compression without cracking or crumbling. All treatments had similar cohesiveness values in the end product and on Day 30 of storage. However, T‐5 and T‐10 might have a uniform and firm texture when compared to the control, T‐1, and ascorbic acid treatments on Day 60 of storage (*p* < 0.05). Moreover, storage caused significant decreases in cohesiveness values of all treatments. Therefore, sucuks might lose uniformity and firmness with storage, irrespective of the addition of terebinth extracts. Chewiness was defined as the amount of chewing needed to break down food to be swallowed. The effect of storage was significant on chewiness values of T‐1 and T‐10 on Day 60 of storage (*p* < 0.05). Even though all treatments had similar values on Day 60 of storage, the addition of terebinth extracts might have had an effect on the toughness of sucuks with a longer storage period. Resilience was defined as the ability of food to regain its original shape following a deformation. The effect of storage was only significant on resilience values of T‐10 with a limited fluctuation (*p* < 0.05). Since all treatments had similar resilience values at each storage period, neither the addition of terebinth extracts nor storage might have a profound effect on resilience values of sucuks (Aguirre et al. 2018; Benli et al. 2024). The primary textural changes in fermented sausage mostly take place during fermentation, when the pH drops and solubilized muscle proteins coagulate to form a gel matrix around the meat and fat particles (Li et al. 2023). Overall, the results indicated that terebinth extracts could be incorporated into sucuks without significantly compromising overall textural quality.

**TABLE 2:** Mean TPA values of sucuks (fermented sausages) manufactured with different levels of terebinth extracts during storage.

Color	Storage period	Control	T‐1	T‐5	T‐10	Ascorbic acid
Hardness (N)	End product	84.33 ± 1.87^aA^	89.70 ± 0.81^aB^	79.33 ± 8.91^aA^	81.34 ± 1.97^aC^	71.15 ± 4.79^aB^
	30 days	100.30 ± 6.78^aA^	94.17 ± 2.89^aB^	82.43 ± 6.80^aA^	98.23 ± 6.89^aB^	91.13 ± 8.61^aAB^
	60 days	119.92 ± 22.08^aA^	133.67 ± 2.52^aA^	99.09 ± 23.57^aA^	119.89 ± 5.43^aA^	110.45 ± 9.37^aA^
Adhesiveness (N. s)	End product	−2.32 ± 0.64^aA^	−2.30 ± 0.36^aB^	−2.62 ± 0.45^aA^	−2.59 ± 0.49^aA^	−2.67 ± 0.03^aA^
	30 days	−2.54 ± 0.13^aA^	−2.40 ± 0.40^aB^	−2.35 ± 0.27^aA^	−2.61 ± 0.15^aA^	−2.53 ± 0.24^aA^
	60 days	−3.70 ± 0.29^aA^	−3.58 ± 0.16^aA^	−3.14 ± 0.45^abA^	−3.02 ± 0.22^abA^	−2.55 ± 0.6^bA^
Cohesivenss	End product	0.469 ± 0.004^aA^	0.452 ± 0.014^aA^	0.487 ± 0.003^aA^	0.500 ± 0.024^aA^	0.499 ± 0.009^aA^
	30 days	0.430 ± 0.023^aAB^	0.414 ± 0.012^aB^	0.437 ± 0.005^aB^	0.440 ± 0.002^aB^	0.444 ± 0.001^aB^
	60 days	0.399 ± 0.015^bB^	0.402 ± 0.004^bB^	0.416 ± 0.002^abC^	0.437 ± 0.011^aB^	0.408 ± 0.008^bC^
Chewiness (N)	End product	21.93 ± 1.48^aA^	21.83 ± 1.06^aB^	23.01 ± 3.55^aA^	22.35 ± 1.09^aB^	20.41 ± 1.35^aA^
	30 days	21.57 ± 3.00^aA^	20.77 ± 2.40^aB^	17.04 ± 1.86^aA^	22.96 ± 1.95^aB^	21.41 ± 0.57^aA^
	60 days	26.62 ± 5.13^aA^	33.13 ± 0.11^aA^	24.20 ± 5.87^aA^	30.80 ± 1.79^aA^	21.63 ± 2.25^aA^
Resilience	End product	0.122 ± 0.005^aA^	0.114 ± 0.001^aA^	0.121 ± 0.006^aA^	0.120 ± 0.004^aAB^	0.118 ± 0.001^aA^
	30 days	0.116 ± 0.009^aA^	0.106 ± 0.006^aA^	0.107 ± 0.001^aA^	0.113 ± 0.004^aB^	0.113 ± 0.007^aA^
	60 days	0.109 ± 0.012^aA^	0.114 ± 0.001^aA^	0.105 ± 0.013^aA^	0.126 ± 0.001^aA^	0.113 ± 0.006^aA^

*Note:* There are significant differences between the mean values (±standard deviation) represented with different lowercase letters on the same row (*p* < 0.05). There are significant differences between the mean values (±standard deviation) represented with different capital letters on the same column for each TPA parameters (*p* < 0.05). T‐1: included 1 mL/kg terebinth extract; T‐5: included 5 mL/kg terebinth extract; T‐10: included 10 mL/kg terebinth extract; and Ascorbic Acid: included 500 mg/kg ascorbic acid.

### 3.6 Biogenic Amines Contents of Fermented Sausages

Table 3 represents mean tryptamine, 2‐phenylethylamine, putrescine, cadaverine, histamine, tyramine, spermidine, and spermine values of fermented sausages (sucuks). The effect of treatments on mean tryptamine contents of sucuks was significant on Day 0 (12.83–30.50 mg/kg dry weight) and Day 60 (12.47–25.14 mg/kg dry weight) (*p* < 0.05). Conversely, all treatments had similar values following the fermentation (Day 3), in the end product, and on Day 30 of the storage ranging from 9.21 to 14.66 mg/kg dry weight. However, the manufacturing/storage period significantly decreased tryptamine contents of T‐1 and T‐5 following the fermentation (Day 3) and significantly increased tryptamine contents of T‐1, T‐5, and T‐10 on Day 60 (*p* < 0.05). The effect of the manufacturing/storage period was not significant on tryptamine contents of the control and ascorbic acid groups, although tryptamine contents numerically decreased during the fermentation (Day 3) and increased during the storage. Wu et al. (2024) reported similar tryptamine contents in fermented and naturally ripened goat meat sausages ranging from 3.25 to 12.80 mg/kg. Likewise, Benli et al. (2024) found decreases and increases in tryptamine contents of sucuks following the fermentation and after 60 days of storage, respectively. Lowering pH through the utilization of starter cultures was associated with lower tryptamine levels in sucuks (Erkmen and Bozkurt 2004). In addition, microbial degradation of tryptamine as a nitrogen source could be the reason for the decreases observed following the fermentation in the current study (Wang, Zhang, et al. 2022). Furthermore, increases in tryptamine levels on Day 60 might suggest a possible resurgence in microbial activity.

**TABLE 3:** Mean biogenic amine values of sucuks (fermented sausages) manufactured with different levels of terebinth extracts during production and storage.

Biogenic amines (mg/kg dry weight)	Manufacturing/Storage period	Control	T‐1	T‐5	T‐10	Ascorbic acid
Tryptamine	0 day	13.85 ± 1.44^bcA^	21.04 ± 1.57^bcA^	30.50 ± 3.60^aA^	12.83 ± 1.85^cB^	22.77 ± 6.02^abA^
	3 days	11.18 ± 1.48^aA^	11.73 ± 1.47^aB^	14.29 ± 0.74^aC^	12.12 ± 1.72^aB^	9.21 ± 1.40^aA^
	End product	14.66 ± 4.31^aA^	12.42 ± 0.74^aB^	11.57 ± 1.71^aC^	10.54 ± 1.24^aB^	11.06 ± 2.34^aA^
	30 days	14.01 ± 4.49^aA^	11.17 ± 2.23^aB^	11.78 ± 1.57^aC^	13.94 ± 0.50^aB^	13.74 ± 4.04^aA^
	60 days	12.47 ± 0.51^bA^	25.14 ± 3.62^aA^	20.99 ± 0.35^abB^	19.46 ± 1.75^bA^	18.20 ± 1.19^bA^
2‐Phenylethylamine	0 days	21.57 ± 1.54^aA^	27.51 ± 1.61^aA^	26.22 ± 3.36^aA^	25.79 ± 1.15^aA^	23.32 ± 1.30^aA^
	3 days	2.32 ± 0.23^aC^	2.12 ± 0.64^aC^	2.23 ± 0.20^aD^	1.68 ± 0.22^aC^	2.41 ± 0.42^aC^
	End product	4.97 ± 1.86^aC^	4.24 ± 0.31^aC^	4.17 ± 0.60^aCD^	4.55 ± 0.43^aBC^	2.76 ± 1.65^aC^
	30 days	11.45 ± 0.34^aB^	14.48 ± 0.53^aB^	12.96 ± 0.53^aB^	9.42 ± 3.29^aB^	10.35 ± 1.62^aB^
	60 days	9.99 ± 3.58^aB^	11.01 ± 4.37^aB^	9.52 ± 4.96^aBC^	11.37 ± 4.85^aB^	14.23 ± 4.83^aB^
Putrescine	0 days	2.17 ± 1.12^aA^	1.32 ± 0.47^aA^	2.60 ± 0.04^aA^	2.17 ± 0.91^aA^	2.51 ± 1.09^aB^
	3 days	1.20 ± 0.15 ^bA^	1.28 ± 0.29^bA^	4.36 ± 1.05^aA^	2.03 ± 1.21^bA^	1.51 ± 0.08^bB^
	End product	1.78 ± 0.77^aA^	1.60 ± 0.05^aA^	2.39 ± 0.21^aA^	2.14 ± 0.32^aA^	1.94 ± 0.16^aB^
	30 days	1.98 ± 0.45^bA^	5.84 ± 2.40^aA^	3.52 ± 0.03^abA^	1.66 ± 0.02^bA^	5.26 ± 0.10^aA^
	60 days	2.45 ± 0.11^aA^	5.63 ± 2.69^aA^	4.17 ± 0.82^aA^	5.58 ± 3.48^aA^	6.11 ± 0.02^aA^
Cadaverine	0 day	4.21 ± 1.45^aA^	2.47 ± 0.21^aA^	3.80 ± 1.77^aA^	2.77 ± 0.13^aA^	5.41 ± 0.72^aA^
	3 days	4.93 ± 0.32^aA^	5.17 ± 0.01^aA^	5.79 ± 1.61^aA^	4.56 ± 1.15^aA^	3.96 ± 0.05^aBC^
	End product	4.15 ± 1.04^aA^	3.45 ± 0.03^aA^	4.01 ± 0.86^aA^	4.77 ± 1.32^aA^	3.25 ± 0.19^aC^
	30 days	4.56 ± 1.10^aA^	4.45 ± 1.45^aA^	2.62 ± 1.25^aA^	2.91 ± 0.50^aA^	3.72 ± 0.37^aBC^
	60 days	2.86 ± 0.19^aA^	4.40 ± 1.52^aA^	3.01 ± 1.10^aA^	2.85 ± 1.32^aA^	4.25 ± 0.02^aB^
Histamine	0 day	7.64 ± 0.20^aA^	7.29 ± 0.95^aA^	7.21 ± 0.03^aA^	7.50 ± 0.33^aA^	7.11 ± 0.04^aAB^
	3 days	6.59 ± 0.25^aA^	6.87 ± 0.49^aA^	7.31 ± 0.66^aA^	6.86 ± 0.12^aA^	7.16 ± 0.18^aAB^
	End product	6.50 ± 0.97^aA^	7.14 ± 0.33^aA^	7.25 ± 0.25^aA^	7.45 ± 0.01^aA^	7.51 ± 0.23^aA^
	30 days	7.33 ± 1.12^aA^	7.40 ± 1.84^aA^	6.56 ± 0.26^aA^	7.18 ± 0.30^aA^	6.80 ± 0.10^aB^
	60 days	6.70 ± 0.30^aA^	8.07 ± 0.87^aA^	8.08 ± 0.60^aA^	7.66 ± 0.46^aA^	7.51 ± 0.24^aA^
Tyramine	0 day	3.18 ± 0.30^aE^	3.07 ± 0.29^aC^	3.33 ± 0.31^aC^	2.77 ± 0.52^aE^	3.44 ± 0.75^aD^
	3 days	22.66 ± 0.83^aD^	23.64 ± 1.91^aB^	24.58 ± 0.39^aB^	22.58 ± 0.31^aD^	22.92 ± 1.22^aC^
	End product	38.16 ± 5.54^aC^	36.94 ± 2.75^aB^	37.20 ± 4.59^aB^	40.54 ± 1.19^aC^	38.67 ± 1.64^aB^
	30 days	47.45 ± 4.50^aB^	61.55 ± 13.89^aA^	68.22 ± 8.60^aA^	49.32 ± 3.68^aB^	49.90 ± 7.84^aA^
	60 days	57.99 ± 0.18^aA^	69.24 ± 3.02^aA^	78.33 ± 12.38^aA^	65.73 ± 0.94^aA^	57.33 ± 0.47^aA^
Spermidine	0 day	4.62 ± 0.15^aBC^	4.49 ± 0.34^aA^	4.50 ± 1.44^aA^	4.81 ± 0.29^aA^	4.27 ± 0.97^aA^
	3 days	5.59 ± 0.32^aAB^	5.20 ± 0.01^aA^	5.22 ± 0.57^aA^	5.04 ± 0.56^aA^	4.83 ± 0.02^aA^
	End product	4.00 ± 0.29^aD^	6.34 ± 3.05^aA^	5.22 ± 1.15^aA^	4.86 ± 0.37^aA^	5.19 ± 0.47^aA^
	30 days	5.96 ± 0.61^aA^	4.86 ± 0.03^aA^	4.39 ± 0.69^aA^	6.84 ± 3.14^aA^	4.59 ± 0.31^aA^
	60 days	4.73 ± 0.56^aBC^	4.78 ± 0.15^aA^	4.71 ± 0.11^aA^	4.87 ± 0.24^aA^	5.17 ± 0.27^aA^
Spermine	0 day	54.88 ± 0.10^aB^	54.64 ± 0.49^aA^	54.09 ± 1.12^aB^	54.85 ± 0.40^aA^	51.08 ± 2.43^aC^
	3 days	52.45 ± 3.04^aB^	53.45 ± 2.40^aA^	57.72 ± 2.84^aB^	55.19 ± 4.48^aA^	53.51 ± 0.88^aBC^
	End product	56.54 ± 0.56^aB^	55.62 ± 0.80^aA^	55.05 ± 0.05^aB^	57.32 ± 3.10^aA^	57.15 ± 1.62^aAB^
	30 days	65.83 ± 2.92^aA^	62.02 ± 8.60^aA^	59.51 ± 2.97^aB^	58.21 ± 1.38^aA^	54.42 ± 1.38^aABC^
	60 days	56.80 ± 0.17^bB^	64.91 ± 4.51^aA^	65.32 ± 1.78^aA^	59.86 ± 1.18^abA^	58.32 ± 1.60^bA^

*Note:* There are significant differences between the mean values (±standard deviation) represented with different lowercase letters on the same row (*p* < 0.05). There are significant differences between the mean values (±standard deviation) represented with different capital letters on the same column for each biogenic amine (*p* < 0.05). T‐1: included 1 mL/kg terebinth extract; T‐5: included 5 mL/kg terebinth extract; T‐10: included 10 mL/kg terebinth extract; and Ascorbic Acid: included 500 mg/kg ascorbic acid.

The effect of treatments on mean 2‐phenylethylamine contents of sucuks was not significant on each of the manufacturing/storage periods. Thus, incorporating terebinth extracts produced similar 2‐phenylethylamine values when compared to the control and ascorbic acid groups on each of the manufacturing/storage periods. However, the manufacturing/storage period had a significant effect on 2‐phenylethylamine contents (*p* < 0.05). The highest 2‐phenylethylamine contents were observed on Day 0, ranging from 21.57 to 27.51 mg/kg dry weight. Fermentation dramatically decreased 2‐phenylethylamine contents of sucuks to a range of 1.68 to 2.41 mg/kg dry weight. Nevertheless, the values numerically increased in the end product and further reached a range of 9.42 to 14.48 mg/kg dry weight on Day 30 (*p* < 0.05), and these values were similar to those on Day 60 of the storage. The initial amount and variety of biogenic amines on a carcass could be determined by the microbial flora present on the surface and the temperature of the surrounding environment (Roseiro et al. 2006). Wang et al. (2021) indicated that inoculating starter cultures significantly decreased biogenic amine contents via synthesizing amine oxidase, which might decompose biogenic amines into aldehydes, hydrogen peroxide, and ammonia gas. The use of starter culture in the current study might explain dramatic decreases in 2‐phenylethylamine values following the fermentation of sucuks.

The effect of treatments on mean putrescine contents of sucuks was significant on Day 3 (1.20–4.36 mg/kg dry weight) and Day 30 (1.66–5.84 mg/kg dry weight) (*p* < 0.05). Conversely, treatments had similar values initially (Day 0), in the end product, and on Day 60 of the storage. In addition, the effect of manufacturing/storage period was only significant on putrescine content of the ascorbic acid group (*p* < 0.05) even though numerical increases were observed in other treatments during the storage period. However, none of the treatments differed significantly from the control in the end product and on Day 60 of the storage. Lower initial putrescine values in sucuks indicated a good hygienic quality of raw material. In addition, the existence of decarboxylating bacteria, including enterobacteria, was reported as an important but not sufficient condition for biogenic amine production (Suvajdzic et al. 2020). In the current study, putrescine values fluctuated in a limited range for all treatments, indicating that terebinth extract can be incorporated into sucuks without a negative effect on putrescine content.

The treatments had no significant effect on the mean cadaverine, histamine, and spermidine values of sucuks on each of the manufacturing/storage periods. In the end products, cadaverine contents ranged from 3.25 to 4.77 mg/kg dry weight, histamine contents ranged from 6.50 to 7.51 mg/kg dry weight, and spermidine contents ranged from 4.00 to 6.34 mg/kg dry weight. The effect of the manufacturing/storage period was significant on cadaverine and histamine contents of the ascorbic acid group and spermidine content of the control group. However, variations in cadaverine, histamine, and spermidine contents were numerically constrained throughout the manufacturing and storage. The results indicated that incorporating terebinth extract might produce similar cadaverine, histamine, and spermidine contents in sucuks.

The treatments had no significant effect on the mean tyramine values of sucuks on each of the manufacturing/storage periods. However, the manufacturing/storage period had a significant effect on tyramine contents (*p* < 0.05). The lowest tyramine contents were observed on Day 0, ranging from 2.77 to 3.44 mg/kg dry weight. Fermentation considerably increased tyramine contents of sucuks to a range of 22.58 to 24.58 mg/kg dry weight. Furthermore, the values increased in the end product and during the storage, reaching a range of 57.33 to 78.33 mg/kg dry weight on Day 60 (*p* < 0.05) of the storage. Tyrosine decarboxylase, the enzyme required to form tyramine, is produced most effectively by lactic acid bacteria. Biogenic amine formation in bacteria is associated with acid resistance or production of metabolic energy, which helps them to survive harsher environmental conditions during the fermentation process (Bargossi et al. 2015). In the current study, increases observed in tyramine values following the fermentation and during the storage might indicate microbial activity of lactic acid bacteria. Komprda et al. (2004) and Benli et al. (2024) have also reported similar increases in tyramine contents of fermented sausages.

The effect of treatments on mean spermine contents of sucuks was only significant on Day 60, ranging from 56.80 to 65.32 mg/kg dry weight (*p* < 0.05). The manufacturing/storage period had a significant effect on spermine contents of the control, T‐5, and ascorbic acid groups (*p* < 0.05). However, the end products of all treatments had similar spermine values, varying between 55.05 and 57.32 mg/kg dry weight. In general, the numerical changes indicated a slight increase in spermine values of sucuks during the storage period. Fermented sausages naturally contain spermine, with only minor fluctuations in its content during manufacturing and storage (Benli et al. 2024). Spermine was also reported to be the most abundant biogenic amine in fermented goat sausage (Wu et al. 2024).

### 3.7 TBARS Values of Fermented Sausages

Table 4 represents mean TBARS values of fermented sausages (sucuks). The effect of treatments on mean TBARS values of sucuks was significant on Day 0 (0.58–1.05 mg malondialdehyde/kg) and Day 3 (1.33–2.16 mg malondialdehyde/kg) during manufacturing (*p* < 0.05). Moreover, the effect of manufacturing/storage period was significant on TBARS values of each treatment during the manufacturing and storage (*p* < 0.05). T‐5 had the lowest TBARS value (0.58 mg malondialdehyde/kg) on Day 0. There were significant increases in TBARS values of all treatments on Day 3 following the fermentation, ranging from 1.33 to 2.16 mg malondialdehyde/kg. However, all treatments produced similar values even though numerical variations were observed in the end product (0.68–1.46 mg malondialdehyde/kg), on Day 30 (0.93–1.88 mg malondialdehyde/kg), and on Day 60 (1.52–1.82 mg malondialdehyde/kg). TBARS values were reported to increase following the fermentation and then decline during the ripening period in Turkish sucuks due to the decomposition into volatile compounds (Akkose et al. 2023; Bozkurt 2006). Furthermore, gradual increases in TBARS values were notified during the storage of bay leaf extract incorporated fermented sausages, similar to the current study (Benli et al. 2024). Nevertheless, numerically lower TBARS values, especially for T‐5, T‐10, and ascorbic acid groups, might be an indication of a decreasing effect of terebinth extracts in the end product and on Day 30 of the storage.

**TABLE 4:** Mean TBARS values of sucuks (fermented sausages) manufactured with different levels of terebinth extracts during production and storage.

	Manufacturing/Storage period	Control	T‐1	T‐5	T‐10	Ascorbic acid
TBARS mg malondialdehyde/kg	0 day	1.05 ± 0.13^aD^	0.81 ± 0.02^bC^	0.58 ± 0.01^cC^	1.01 ± 0.05^aB^	1.03 ± 0.08^aB^
	3 days	1.33 ± 0.01^cCD^	1.56 ± 0.32^bcB^	1.88 ± 0.03^abA^	1.64 ± 0.17^bcA^	2.16 ± 0.02^aA^
	End product	1.46 ± 0.18^aB^	1.25 ± 0.01^aBC^	0.68 ± 0.08^aBC^	0.77 ± 0.25^aB^	0.93 ± 0.34^aB^
	30 days	1.88 ± 0.10^aA^	1.18 ± 0.25^aBC^	0.93 ± 0.01^aB^	1.02 ± 0.38^aB^	1.05 ± 0.38^aB^
	60 days	1.52 ± 0.14^aB^	1.63 ± 0.01^aA^	1.68 ± 0.28^aA^	1.63 ± 0.11^aA^	1.82 ± 0.27^aA^

*Note:* There are significant differences between the mean values (±standard deviation) represented with different lowercase letters on the same row (*p* < 0.05). There are significant differences between the mean values (±standard deviation) represented with different capital letters on the same column (*p* < 0.05). T‐1: included 1 mL/kg terebinth extract; T‐5: included 5 mL/kg terebinth extract; T‐10: included 10 mL/kg terebinth extract; and Ascorbic Acid: included 500 mg/kg ascorbic acid.

## 4 Conclusion

Improving quality and safety of fermented sausages is an important issue due to accumulation of biogenic amines in the fermented foods. In this study, an ultrasound‐assisted extract of *Pistacia terebinthus* L. using 70% ethanol was selected as a natural source of antioxidants and phytochemicals due to its higher total phenolic content and DPPH value. The obtained terebinth extract was incorporated into sucuks without having a negative effect on the quality. The results indicated that 1, 5, and 10 mg/L terebinth extract could be incorporated into sucuks without extensively compromising moisture content, pH, titratable acidity, color values, TPA values, and TBARS values of the samples when compared to the control and ascorbic acid group. Terebinth extract caused no significant decline in biogenic amine accumulations of the sucuk samples at the current concentrations. Nevertheless, all treatments had similar biogenic amine values in the end products. Although the storage had a significant effect on biogenic amine values of some treatments, significant differences were only observed in tryptamine and spermine values of the samples at the end of the storage. Consequently, terebinth extract might be used in the commercial production of fermented sausages to improve their antioxidant and phytochemical characteristics without having a negative effect on the overall quality. The future studies should take into account the incorporation of terebinth extracts at higher concentrations or in powdered form.

## Author Contributions

**Hakan Benli:** conceptualization (lead), formal analysis (lead), funding acquisition (lead), investigation (equal), methodology (equal), project administration (lead), resources (lead), supervision (lead), validation (equal), visualization (lead), writing – original draft (lead), writing – review and editing (lead). **Özge Tetik:** conceptualization (equal), formal analysis (equal), funding acquisition (supporting), investigation (equal), methodology (equal), project administration (supporting), resources (equal), validation (equal), writing – original draft (supporting), writing – review and editing (supporting).

## Conflicts of Interest

The authors declare no conflicts of interest.

## Data Availability

The relevant data will be supplied upon reasonable request.

## References

[fsn370853-bib-0001] Abdullakasim, P. , S. Songchitsomboon , M. Techagumpuch , N. Balee , P. Swatsitang , and P. Sungpuag . 2007. “Antioxidant Capacity, Total Phenolics and Sugar Content of Selected Thai Health Beverages.” International Journal of Food Sciences and Nutrition 58, no. 1: 77–85. 10.1080/09637480601140946. 17415958

[fsn370853-bib-0002] Abril, B. , R. Bou , J. V. Garcia‐Perez , and J. Benedito . 2023. “Role of Enzymatic Reactions in Meat Processing and Use of Emerging Technologies for Process Intensification.” Food 12, no. 10: 1940. 10.3390/foods12101940. PMC10217372 37238758

[fsn370853-bib-0003] Agcam, E. , A. Akyildiz , S. Kamat , and V. M. Balasubramaniam . 2021. “Bioactive Compounds Extraction From the Black Carrot Pomace With Assistance of High Pressure Processing: An Optimization Study.” Waste and Biomass Valorization 12, no. 11: 5959–5977. 10.1007/s12649-021-01431-z.

[fsn370853-bib-0004] Aguirre, M. E. , C. M. Owens , R. K. Miller , and C. Z. Alvarado . 2018. “Descriptive Sensory and Instrumental Texture Profile Analysis of Woody Breast in Marinated Chicken.” Poultry Science 97, no. 4: 1456–1461. 10.3382/ps/pex428. 29438548

[fsn370853-bib-0005] Akkose, A. , S. S. Ogras , M. Kaya , and G. Kaban . 2023. “Microbiological, Physicochemical and Sensorial Changes During the Ripening of Sucuk, a Traditional Turkish Dry‐Fermented Sausage: Effects of Autochthonous Strains, Sheep Tail Fat and Ripening Rate.” Fermentation‐Basel 9, no. 6: 558. 10.3390/fermentation9060558.

[fsn370853-bib-0006] Akyuz, M. , L. Yabo‐Dambagi , T. Kilic , and A. Cakir . 2022. “Antidiabetic, Neuroprotective and Antioxidant Potentials of Different Parts of *Pistacia terebinthus* Fruits.” South African Journal of Botany 147: 443–456. 10.1016/j.sajb.2022.01.040.

[fsn370853-bib-0007] Alberto, M. R. , M. E. Arena , and M. C. Manca de Nadra . 2007. “Putrescine Production From Agmatine by *Lactobacillus hilgardii* : Effect of Phenolic Compounds.” Food Control 18, no. 8: 898–903. 10.1016/j.foodcont.2006.05.006.

[fsn370853-bib-0008] Alothman, M. , R. Bhat , and A. A. Karim . 2009. “Antioxidant Capacity and Phenolic Content of Selected Tropical Fruits From Malaysia, Extracted With Different Solvents.” Food Chemistry 115, no. 3: 785–788. 10.1016/j.foodchem.2008.12.005.

[fsn370853-bib-0009] Altemimi, A. , N. Lakhssassi , A. Baharlouei , D. G. Watson , and D. A. Lightfoot . 2017. “Phytochemicals: Extraction, Isolation, and Identification of Bioactive Compounds From Plant Extracts.” Plants (Basel) 6, no. 4: 42. 10.3390/plants6040042. 28937585 PMC5750618

[fsn370853-bib-0010] Bakırel, T. , S. Şener , U. Bakırel , O. Keleş , G. Şennazlı , and A. Gürel . 2003. “The Investigation of the Effects of *Pistacia terebinthus* L. Upon Experimentally Induced Hypercholesterolemia and Atherosclerosis in Rabbits.” Turkish Journal of Veterinary and Animal Sciences 27, no. 6: 6.

[fsn370853-bib-0011] Bargossi, E. , G. Tabanelli , C. Montanari , et al. 2015. “Tyrosine Decarboxylase Activity of Enterococci Grown in Media With Different Nutritional Potential: Tyramine and 2‐Phenylethylamine Accumulation and tyrDC Gene Expression.” Frontiers in Microbiology 6: 259. 10.3389/fmicb.2015.00259. 25914676 PMC4392317

[fsn370853-bib-0012] Becker, T. 2002. “Defining Meat Quality.” In Meat Processing: Improving Quality, edited by J. Kerry , J. Kerry , and D. Ledward , 464. CRC Press.

[fsn370853-bib-0013] Benli, H. 2017. “Some Chemical Characteristics of Sucuk and Salami Samples Available at Retail in Adana.” Turkish Journal of Agriculture, Food Science and Technology 5, no. 11: 1307. 10.24925/turjaf.v5i11.1307–1311.1357.

[fsn370853-bib-0014] Benli, H. , and E. Barutcu . 2021. “Sequential Use of Real‐Time Polymerase Chain Reaction and Enzyme‐Linked Immunosorbent Assay Techniques Verifies Adulteration of Fermented Sausages With Chicken Meat.” Animal Bioscience 34, no. 12: 1995–2002. 10.5713/ab.21.0139. 34237920 PMC8563240

[fsn370853-bib-0015] Benli, H. , P. Şahin , and E. Ağçam . 2024. “Incorporating Bay Leaf Extract ( *Laurus nobilis* L.) and Determining the Quality Attributes of Turkish Fermented Sausage (Sucuk).” Food Science & Nutrition 12, no. 4: 2473–2487. 10.1002/fsn3.3929. 38628223 PMC11016401

[fsn370853-bib-0016] Bover‐Cid, S. , M. Izquierdo‐Pulido , and M. C. Vidal‐Carou . 2001. “Changes in Biogenic Amine and Polyamine Contents in Slightly Fermented Sausages Manufactured With and Without Sugar.” Meat Science 57, no. 2: 215–221. 10.1016/S0309-1740(00)00096-6. 22061366

[fsn370853-bib-0017] Bozkurt, H. 2006. “Investigation of the Effect of Sumac Extract and BHT Addition on the Quality of Sucuk (Turkish Dry‐Fermented Sausage).” Journal of the Science of Food and Agriculture 86, no. 5: 849–856. 10.1002/jsfa.2431.

[fsn370853-bib-0018] Bozorgi, M. , Z. Memariani , M. Mobli , M. H. Salehi Surmaghi , M. R. Shams‐Ardekani , and R. Rahimi . 2013. “Five Pistacia Species ( *P. vera* , *P. atlantica* , *P. terebinthus* , *P. khinjuk*, and *P. lentiscus* ): A Review of Their Traditional Uses, Phytochemistry, and Pharmacology.” ScientificWorldJournal 2013: 219815. 10.1155/2013/219815. 24453812 PMC3876903

[fsn370853-bib-0019] Calnan, H. , R. H. Jacob , D. W. Pethick , and G. E. Gardner . 2016. “Production Factors Influence Fresh Lamb Longissimus Colour More Than Muscle Traits Such as Myoglobin Concentration and pH.” Meat Science 119: 41–50. 10.1016/j.meatsci.2016.04.009. 27129082

[fsn370853-bib-0020] Cardona, M. , D. Izquierdo , J. M. Barat , and I. Fernández‐Segovia . 2023. “Intrinsic and Extrinsic Attributes That Influence Choice of Meat and Meat Products: Techniques Used in Their Identification.” European Food Research and Technology 249, no. 10: 2485–2514. 10.1007/s00217-023-04301-1.

[fsn370853-bib-0021] Chemat, F. , N. Rombaut , A. G. Sicaire , A. Meullemiestre , A. S. Fabiano‐Tixier , and M. Abert‐Vian . 2017. “Ultrasound Assisted Extraction of Food and Natural Products. Mechanisms, Techniques, Combinations, Protocols and Applications. A Review.” Ultrasonics Sonochemistry 34: 540–560. 10.1016/j.ultsonch.2016.06.035. 27773280

[fsn370853-bib-0022] Dai, J. , and R. J. Mumper . 2010. “Plant Phenolics: Extraction, Analysis and Their Antioxidant and Anticancer Properties.” Molecules 15, no. 10: 7313–7352. 10.3390/molecules15107313. 20966876 PMC6259146

[fsn370853-bib-0023] Demeyer, D. , and L. Stahnke . 2002. “Quality Control of Fermented Meat Products.” In Meat Processing: Improving Quality, edited by J. Kerry and D. Ledward , 464. CRC Press.

[fsn370853-bib-0024] Eerola, S. , R. Hinkkanen , E. Lindfors , and T. Hirvi . 1993. “Liquid‐Chromatographic Determination of Biogenic‐Amines in Dry Sausages.” Journal of AOAC International 76, no. 3: 575–577. 10.1093/jaoac/76.3.575. 8318851

[fsn370853-bib-0025] Erkmen, O. , and H. Bozkurt . 2004. “Quality Characteristics of Retailed Sucuk (Turkish Dry‐Fermented Sausage).” Food Technology and Biotechnology 42, no. 1: 63–69.

[fsn370853-bib-0026] Gençcelep, H. , G. Kaban , M. I. Aksu , F. Oz , and M. Kaya . 2008. “Determination of Biogenic Amines in Sucuk.” Food Control 19, no. 9: 868–872. 10.1016/j.foodcont.2007.08.013.

[fsn370853-bib-0027] Gökalp, H. Y. , M. Kaya , Y. Tülek , and Ö. Zorba . 2001. Et ve ürünlerinde Kalite Kontrolü ve Laoratuvar Uygulama Klavuzu. Atatürk Üniversitesi Ziraat Fakültesi Oset Tesisi (in Turkish).

[fsn370853-bib-0028] Haminiuk, C. W. , M. S. Plata‐Oviedo , G. de Mattos , S. T. Carpes , and I. G. Branco . 2014. “Extraction and Quantification of Phenolic Acids and Flavonols From Eugenia Pyriformis Using Different Solvents.” Journal of Food Science and Technology 51, no. 10: 2862–2866. 10.1007/s13197-012-0759-z. 25328239 PMC4190214

[fsn370853-bib-0029] Kamiloglu, S. , T. Ozdal , S. Bakir , and E. Capanoglu . 2022. “Bioaccessibility of Terebinth ( *Pistacia terebinthus* L.) Coffee Polyphenols: Influence of Milk, Sugar and Sweetener Addition.” Food Chemistry 374: 131728. 10.1016/j.foodchem.2021.131728. 34891090

[fsn370853-bib-0030] Kargozari, M. , S. Moini , A. A. Basti , et al. 2014. “Development of Turkish Dry‐Fermented Sausage (Sucuk) Reformulated With Camel Meat and Hump Fat and Evaluation of Physicochemical, Textural, Fatty Acid and Volatile Compound Profiles During Ripening.” LWT ‐ Food Science and Technology 59, no. 2: 849–858. 10.1016/j.lwt.2014.05.033.

[fsn370853-bib-0031] Kaya, F. , and A. Özer . 2015. “Characterization of Extracted Oil From Seeds of Terebinth (*Pistacia terebinthus* L.) Growing Wild in Turkey.” Turkish Journal of Science and Technology 10, no. 1: 49–57.

[fsn370853-bib-0032] Klimczak, I. , M. Malecka , M. Szlachta , and A. Gliszczynska‐Swiglo . 2007. “Effect of Storage on the Content of Polyphenols, Vitamin C and the Antioxidant Activity of Orange Juices.” Journal of Food Composition and Analysis 20, no. 3–4: 313–322. 10.1016/j.jfca.2006.02.012.

[fsn370853-bib-0033] Komprda, T. , D. Smela , P. Pechova , L. Kalhotka , J. Stencl , and B. Klejdus . 2004. “Effect of Starter Culture, Spice Mix and Storage Time and Temperature on Biogenic Amine Content of Dry Fermented Sausages.” Meat Science 67, no. 4: 607–616. 10.1016/j.meatsci.2004.01.003. 22061810

[fsn370853-bib-0034] Kumar, A. , N. P , M. Kumar , et al. 2023. “Major Phytochemicals: Recent Advances in Health Benefits and Extraction Method.” Molecules 28, no. 2: 887. 10.3390/molecules28020887. 36677944 PMC9862941

[fsn370853-bib-0035] Kurcubic, V. S. , P. Z. Maskovic , J. M. Vujic , et al. 2014. “Antioxidant and Antimicrobial Activity of Kitaibelia Vitifolia Extract as Alternative to the Added Nitrite in Fermented Dry Sausage.” Meat Science 97, no. 4: 459–467. 10.1016/j.meatsci.2014.03.012. 24769144

[fsn370853-bib-0036] Lee, J.‐Y. , Y.‐g. Kim , J.‐Y. Her , M. K. Kim , and K.‐G. Lee . 2018. “Reduction of Biogenic Amine Contents in Fermented Soybean Paste Using Food Additives.” LWT ‐ Food Science and Technology 98: 470–476. 10.1016/j.lwt.2018.09.015.

[fsn370853-bib-0037] Lee, Y. H. , B. H. Kim , J. H. Kim , W. S. Yoon , S. H. Bang , and Y. K. Park . 2007. “CadC Has a Global Translational Effect During Acid Adaptation in *Salmonella enterica* Serovar Typhimurium.” Journal of Bacteriology 189, no. 6: 2417–2425. 10.1128/Jb.01277-06. 17209022 PMC1899395

[fsn370853-bib-0038] Li, Y. Z. , Y. K. Geng , D. Shi , R. T. Li , J. Tang , and S. L. Lu . 2023. “Impact of *Coreopsis tinctoria* Nutt. Essential Oil Microcapsules on the Formation of Biogenic Amines and Quality of Smoked Horsemeat Sausage During Ripening.” Meat Science 195: 109020. 10.1016/j.meatsci.2022.109020. 36334510

[fsn370853-bib-0039] Manzoor, A. , A. Haque , S. Ahmad , and D. L. Hopkins . 2023. “Incorporation of Betel Leaf Extract Provides Oxidative Stability and Improves Phytochemical, Textural, Sensory and Antimicrobial Activities of Buffalo Meat Sausages.” Meat Science 200: 109157. 10.1016/j.meatsci.2023.109157. 36913796

[fsn370853-bib-0040] Mielnik, M. B. , E. Olsen , G. Vogt , D. Adeline , and G. Skrede . 2006. “Grape Seed Extract as Antioxidant in Cooked, Cold Stored Turkey Meat.” LWT‐ Food Science and Technology 39, no. 3: 191–198. 10.1016/j.lwt.2005.02.003.

[fsn370853-bib-0041] Muniz‐Marquez, D. B. , G. C. Martinez‐Avila , J. E. Wong‐Paz , R. Belmares‐Cerda , R. Rodriguez‐Herrera , and C. N. Aguilar . 2013. “Ultrasound‐Assisted Extraction of Phenolic Compounds From *Laurus nobilis* L. and Their Antioxidant Activity.” Ultrasonics Sonochemistry 20, no. 5: 1149–1154. 10.1016/j.ultsonch.2013.02.008. 23523026

[fsn370853-bib-0042] Naczk, M. , and F. Shahidi . 2006. “Phenolics in Cereals, Fruits and Vegetables: Occurrence, Extraction and Analysis.” Journal of Pharmaceutical and Biomedical Analysis 41, no. 5: 1523–1542. 10.1016/j.jpba.2006.04.002. 16753277

[fsn370853-bib-0043] Nielsen, S. S. 2003. Food Analysis. 3rd ed. Kluwer Academic/Plenum Publishers.

[fsn370853-bib-0044] Ockerman, H. W. 1985. Quality Control of Post‐Mortem Muscle Tissue. Department of Animal Science, Ohio State University and the Ohio Agricultural Research and Development Center. https://books.google.com.tr/books?id=DoauugAACAAJ.

[fsn370853-bib-0045] Ott, L. , and M. Longnecker . 2001. An Introduction to Statistical Methods and Data Analysis. 5th ed. Duxbury.

[fsn370853-bib-0046] Ozcan, M. 2004. “Characteristics of Fruit and Oil of Terebinth (*Pistacia terebinthus* L) Growing Wild in Turkey.” Journal of the Science of Food and Agriculture 84: 517–520. 10.1002/jsfa.1632.

[fsn370853-bib-0047] Plaskova, A. , and J. Mlcek . 2023. “New Insights of the Application of Water or Ethanol‐Water Plant Extract Rich in Active Compounds in Food.” Frontiers in Nutrition 10: 1118761. 10.3389/fnut.2023.1118761. 37057062 PMC10086256

[fsn370853-bib-0048] Rhee, J. E. , J. H. Rhee , P. Y. Ryu , and S. H. Choi . 2002. “Identification of the cadBA Operon From Vibrio Vulnificus and Its Influence on Survival to Acid Stress.” FEMS Microbiology Letters 208, no. 2: 245–251. 11959444 10.1111/j.1574-6968.2002.tb11089.x

[fsn370853-bib-0049] Roseiro, C. , C. Santos , M. Sol , L. Silva , and I. Fernandes . 2006. “Prevalence of Biogenic Amines During Ripening of a Traditional Dry Fermented Pork Sausage and Its Relation to the Amount of Sodium Chloride Added.” Meat Science 74, no. 3: 557–563. 10.1016/j.meatsci.2006.03.030. 22063060

[fsn370853-bib-0050] Ruiz‐Capillas, C. , and A. M. Herrero . 2019. “Impact of Biogenic Amines on Food Quality and Safety.” Food 8, no. 2: 62. 10.3390/foods8020062. PMC6406683 30744001

[fsn370853-bib-0051] Siriken, B. , O. Cadirci , G. Inat , C. Yenisey , M. Serter , and M. Ozdemir . 2009. “Some Microbiological and Pysico‐Chemical Quality of Turkish Sucuk (Sausage).” Journal of Animal and Veterinary Advances 8, no. 10: 2027–2032.

[fsn370853-bib-0052] Spano, G. , P. Russo , A. Lonvaud‐Funel , et al. 2010. “Biogenic Amines in Fermented Foods.” European Journal of Clinical Nutrition 64, no. Suppl 3: S95–S100. 10.1038/ejcn.2010.218. 21045859

[fsn370853-bib-0053] Sun, X. Y. , B. Du , L. H. Zhao , et al. 2020. “The Effect of Different Starter Cultures on Biogenic Amines and Quality of Fermented Mutton Sausages Stored at 4 and 20°C Temperatures.” Food Science & Nutrition 8, no. 8: 4472–4483. 10.1002/fsn3.1748. 32884727 PMC7455928

[fsn370853-bib-0054] Suvajdzic, B. , T. Tasic , V. Teodorovic , et al. 2020. “Biogenic Amine Content During the Production and Ripening of Sremski Kulen, Serbian Traditional Dry Fermented Sausage.” Animal Science Journal 91, no. 1: 13466. 10.1111/asj.13466. 33043554

[fsn370853-bib-0055] Swider, O. , M. L. Roszko , and M. Wojcicki . 2023. “The Inhibitory Effects of Plant Additives on Biogenic Amine Formation in Fermented Foods—A Review.” Critical Reviews in Food Science and Nutrition 64: 12935–12960. 10.1080/10408398.2023.2258964. 37724793

[fsn370853-bib-0056] Topçu, G. , M. Ay , A. Bilici , C. Sarıkürkcü , M. Öztürk , and A. Ulubelen . 2007. “A New Flavone From Antioxidant Extracts of *Pistacia terebinthus* .” Food Chemistry 103, no. 3: 816–822. 10.1016/j.foodchem.2006.09.028.

[fsn370853-bib-0057] van de Guchte, M. , P. Serror , C. Chervaux , T. Smokvina , S. D. Ehrlich , and E. Maguin . 2002. “Stress Responses in Lactic Acid Bacteria.” Antonie van Leeuwenhoek International Journal of General and Molecular Microbiology 82, no. 1–4: 187–216. 10.1023/A:1020631532202. 12369188

[fsn370853-bib-0058] Wang, D. , G. Hu , H. Wang , et al. 2021. “Effect of Mixed Starters on Proteolysis and Formation of Biogenic Amines in Dry Fermented Mutton Sausages.” Food 10, no. 12: 2939. 10.3390/foods10122939. PMC8700690 34945490

[fsn370853-bib-0059] Wang, H. P. , H. W. Zhang , S. T. Liu , L. G. Qin , Q. Chen , and B. H. Kong . 2022. “Analysis of Biogenic Amine in Dry Sausages Collected From Northeast China: From the Perspective of Free Amino Acid Profile and Bacterial Community Composition.” Food Research International 162: 112084. 10.1016/j.foodres.2022.112084. 36461333

[fsn370853-bib-0060] Wang, Q. , K. Y. Liu , J. H. Zhang , J. S. An , C. Zhang , and T. Chen . 2022. “Research Progress of Biogenic Amines in Fermented Sausages: A Review.” International Food Research Journal 29, no. 2: 223–235. 10.47836/ifrj.29.2.01.

[fsn370853-bib-0061] Wang, Y. , F. Li , H. Zhuang , et al. 2015. “Effects of Plant Polyphenols and α‐Tocopherol on Lipid Oxidation, Residual Nitrites, Biogenic Amines, and N‐Nitrosamines Formation During Ripening and Storage of Dry‐Cured Bacon.” LWT‐ Food Science and Technology 60, no. 1: 199–206. 10.1016/j.lwt.2014.09.022.

[fsn370853-bib-0062] Wendakoon, C. N. , and M. Sakaguchi . 1995. “Inhibition of Amino Acid Decarboxylase Activity of *Enterobacter aerogenes* by Active Components in Spices.” Journal of Food Protection 58, no. 3: 280–283. 10.4315/0362-028X-58.3.280. 31137282

[fsn370853-bib-0063] Wu, S. H. , Y. Niu , J. Wang , X. Dao , Y. Q. Lin , and J. Chen . 2024. “The Influence of Different Levels of Sodium Chloride, Sodium Nitrite, and Glucose on Biogenic Amines and Microbial Communities in Fermented Goat Meat Sausage.” Food 13, no. 6: 817. 10.3390/foods13060817. PMC10969002 38540808

[fsn370853-bib-0064] Xu, Z. , J. Chang , J. Zhou , et al. 2023. “Characterization and Mechanism of Tea Polyphenols Inhibiting Biogenic Amine Accumulation in Marinated Spanish Mackerel.” Food 12, no. 12: 2347. 10.3390/foods12122347. PMC10297285 37372558

[fsn370853-bib-0065] Yin, M.‐c. , and W.‐s. Cheng . 2003. “Antioxidant and Antimicrobial Effects of Four Garlic‐Derived Organosulfur Compounds in Ground Beef.” Meat Science 63, no. 1: 23–28. 10.1016/S0309-1740(02)00047-5. 22061980

[fsn370853-bib-0066] Zhang, Y. , J. L. Jia , Q. Qian , et al. 2024. “Effect of Isolated Bacteria on Nitrite Degradation and Quality of Sichuan Dry Sausages.” LWT‐ Food Science and Technology 212: 117039. 10.1016/j.lwt.2024.117039.

